# Rheumatoid arthritis chondrocytes produce increased levels of pro-inflammatory proteins

**DOI:** 10.1016/j.ocarto.2022.100235

**Published:** 2022-01-21

**Authors:** Karin Önnheim, Shan Huang, Alexander Strid Holmertz, Sofia Andersson, Erik Lönnblom, Charlotte Jonsson, Rikard Holmdahl, Inger Gjertsson

**Affiliations:** a Dep of Rheumatology and Inflammation Research, Institute of Medicine, Gothenburg University, Sweden; b Medical Inflammation Research, Dept of Medical Biochemistry and Biophysics, the Karolinska Institute, Stockholm, Sweden

**Keywords:** Chondrocyte, Cytokine, Rheumatoid arthritis

## Abstract

**Objective:**

To investigate whether articular chondrocytes from rheumatoid arthritis (RA) patients have acquired a proinflammatory phenotype.

**Method:**

Articular cartilage explants from RA patients and healthy controls (HC) were cultured with or without interleukin (IL)-1β for two weeks. Protein levels of cytokines and metalloproteinases (MMPs) in the supernatant were measured by LUMINEX, mRNA with qPCR and nitrogen oxide (NO) levels with Griess assay.

**Results:**

Within 24 ​h after culture, cartilage explants from RA spontaneously produced MMP-1 and MMP-13, and matrix components (aggrecan and collagen type IV) were released. In addition, the RA explants released higher levels of tumor necrosis factor, interferon-γ, IL-33, IL-18, vascular endothelial growth factor-A, IL-6 but not IL-8, and granulocyte-macrophage colony-stimulating factor (GM-CSF) as compared with HC. During two weeks of incubation the higher levels did not diminish. IL-1β stimulation further increased the levels of IL-6, IL-8 and GM-CSF, mainly in RA explants, and induced increased levels of NO in the supernatant from both HC and RA explants, as a result of chondrocyte activation.

**Conclusions:**

RA chondrocytes are activated with a proinflammatory profile involving the production of cytokines as well as MMP-1 and MMP-13, that can lead to release of matrix molecules after activation, which suggests that the chondrocytes have a proinflammatory phenotype and thereby an active role in the pathogenesis.

## 1 Introduction

Rheumatoid arthritis (RA) is characterized by chronic inflammation of cartilaginous diarthrodial joints leading to bone and cartilage degradation and erosions. The inflammatory cells in the synovia are pathogenic by producing proinflammatory cytokines and enzymes driving the chronic inflammatory process. Inhibition of pathogenic cytokines such as tumor necrosis factor (TNF) and interleukin (IL)-6 are today well-established therapies [1]. The chronic active inflammation in the joints is characterized by the transformation of the synovia into a pannus tissue which is composed of activated cells, mainly macrophages and fibroblasts. The inflammation leads to the activation of osteoclasts and severe erosions of joint proximal bone. The joint cartilage is destabilized and show signs of erosions on the surface but are seldom completely eroded. With time the joints are deformed leading to severe dysfunction and chronic pain.

The chondrocytes, being the only cell in articular cartilage, are responsible for maintaining the extracellular cartilage matrix (ECM), but during joint inflammation the metabolic balance is disturbed resulting in cartilage loss [2]. Cartilage is believed to be damaged by several different mediators such as proinflammatory factors released from inflammatory synoviocytes, e.g. IL-1β, TNF and IL-6, proteases such as matrix metalloproteinases (MMP) and nitrogen oxide (NO) [3,4]. Many studies on articular chondrocytes have been performed on cartilage from osteoarthritis (OA) patients. If and how human articular chondrocytes can contribute to the cartilage destruction in RA due to cytokine and MMP production is not well investigated.

Articular cartilage is an avascular tissue and the limited supply of both oxygen and nutrients is through diffusion resulting in a slow regenerative ability. Nutrition comes from the surrounding synovial fluid by diffusion facilitated by varying compression of the cartilage tissue [5]. The articular cartilage normally has a low turnover where the major protein, collagen type II, has a half-life of over 100 years while the proteoglycan aggrecan has been estimated to 3–24 years [6,7]. Chondrocytes constitute only 1–2% of the matrix and produce a low but steady level of components that supports the ECM during normal conditions. In a chronic inflammatory disease such as RA, the equilibrium between anabolism and catabolism is disturbed and the joint tissue is degraded faster than it is rebuilt [2]. Degradation of ECM is orchestrated by both MMPs and a disintegrin and metalloproteinase with thrombospondin motifs (ADAMTS) that target the two major components in articular cartilage, collagen type II and aggrecan [8]. Leucocytes attracted to the inflammation and surrounding synovial fibroblast all produce proinflammatory cytokines such as TNF and IL-1β, that can alter the phenotype of the chondrocytes by suppressing synthesis of collagen type II and aggrecan, stimulate expression of proteases that can break down ECM, inhibit chondrocyte proliferation and cause production of matrix components not present in normal cartilage [4,9].

Studies regarding the contribution by chondrocytes to the synthesis of proinflammatory cytokines are scarce and mainly focused on chondrocytes from OA patients [10]. To the best of our knowledge, very little is known about production of cytokines and other proinflammatory mediators from chondrocytes in explant from RA patients. We hypothesized that human articular chondrocytes from patients with RA contribute to local inflammation in the joint, which is further enhanced by addition of IL-1β. The aim of this study was to investigate this hypothesis by comparing the production of proinflammatory proteins and cartilage components from explants derived from RA patients and healthy controls.

## 2 Material and methods

### 2.1 Cartilage sampling

RA patients undergoing prosthetic hip or knee replacement surgery and patients with fractured femur head (HC) were enrolled in this study to collect human articular cartilage. The study was approved by the ethical committee (Dnr 334-15, 2015-05-18; T1075-17, 2017-12-18 2019–04373 2019-09-11) and all patients signed an informed written consent before entering the study. Demographic characteristics regarding patients in the study can be seen in Table 1.

**Table 1:** Demographic characteristics of patients included in the study.

	HC (n ​= ​10)	RA (n ​= ​8)
Age at time for surgery (median, min; max)	76 (61; 93)	67 (49; 75)
Female n, (%)	8 (80%)	6 (75%)
Disease duration at time for surgery (median, min; max)		10 (2; 17)
RF positivity n, (%)	N/A	7 (88%)
Anti-rheumatic treatment		
DMARDs		
Methotrexate n, (%)	N/A	6 (75%)
Biologics (infliximab) n, (%)	N/A	2 (25%)
Prednisolon	N/A	1 (12%)
No DMARDs or prednisolon	N/A	1 (12)

Healthy controls (HC), rheumatoid arthritis (RA), Not Applicable (N/A), rheumatoid factor (RF), disease modifying anti rheumatoid drugs (DMARDs).

### 2.2 Explants cultures

After surgery, cartilage samples were placed in phosphate buffered saline (PBS) and stored at 4 ​°C until further handling. Full thickness cartilage was carved of within 24 ​h after surgery and explants, 4 ​mm in diameter, were punched out using a biopsy punch. Explants were washed in PBS before *in vitro* cultivation in a Nunclon Delta Surface 96 wells plate at 37 ​°C with 5% CO_2_. 200 ​μl cultivation medium Dulbecco's Modified Eagle Medium (DMEM) (Gibco by Life Technologies, Paisley, UK) supplemented with 10% fetal bovine serum (Gibco by Life Technologies, Paisley, UK), 1% ascorbic acid and 1% penicillin and streptomycin was added to each well. Stimulation was performed with IL-1β (0.01 ​μg/ml) (Peprotech, Stockholm, Sweden) and explants in cultivation medium without stimulation served as medium control. Medium was changes for all samples at day 0, 1, 3, 6, 9, 12 and 14 and the supernatants were harvested. Day 0 samples was obtained after 16–24 ​h incubation in culture medium and serve as baseline before IL-1β stimulation. All stimulations were performed on duplicate explants (with exception for day 0 that are based on the medium value from five duplicates) from each patient/healthy control and the supernatants from these duplicates were pooled and frozen immediately at −80 ​°C until further analysis. Values presented for day 0 are based on five measurements from the pooled duplicates, and the median value for each patient and each time point is presented.

### 2.3 Cytokine and protein levels in supernatants

A Human Premixed Multi-Analyte Magnetic Luminex Assay (R&D systems, Minneapolis, MN, USA) was used to measure the protein levels of granulocyte-macrophage colony-stimulating factor (GM-CSF) GM-CSF, C-X-C Motif Chemokine Ligand 8 (CXCL8)/IL-8, IL-33, TNF, interferon-γ, IL-6, IL-18, ADAMTS13, MMP-1 (propeptide), MMP-8, MMP-13 (propeptide), vascular endothelial growth factor A (VEGF-A), collagen IV a1 and aggrecan in supernatants according to the manufacturer's protocol. Briefly, a 1:3 dilution of supernatants from day 0, 3, 6, 9, 14 together with standards were mixed with magnetic beads and incubated for 2 ​h on a shaker in room temperature. After washing, a biotinylated antibody mix was added to all samples and incubated for 1 ​h. The plates were washed, and streptavidin-PE was added to all wells and incubated for 30 ​min. Using a Bio-Plex 200 system (Bio-Rad Laboratories, Hercules, CA, USA) the magnetic beads-antibody complexes was quantified and analyzed with a five parametric logistic standard curve, which was used for interpolation, recovery rate 80–120.

### 2.4 Chondrocyte qPCR

Chondrocytes were isolated at day 0 from ECM derived from n ​= ​6 HC and n ​= ​5 RA by treatment with 0.3% collagenase (Worthington Biochemical Corporation, Lakewook, CA, USA) as previously described [11]. The isolated chondrocytes were frozen in fetal bovine serum with 10% DMSO (Sigma-Aldrich, Stockholm, Sweden) and stored at −150^○^C until further use. RNA was isolated using a RNeasy Mini kit (Qiagen, Hilden, Germany) and cDNA, 20 μl/reaction, was synthesized using a High-Capacity cDNA Reverse Transcription Kit (Applied Biosystems, Waltham, MA, USA), both according to the manufacturer's instructions.

Gene expression was investigated using quantitative real-time PCR (qRT-PCR) with TaqMan™ Universal Master Mix II with UNG (Applied Biosystems, Waltham, MA, USA) and primers for IL-6 (Hs00174131_m1), MMP-13 (Hs00942584_m1), IL-8 (Hs00174103_m1), TNF (Hs00174128_m1) and glyceraldehyde 3-phosphate dehydrogenase (GAPDH) (Hs03929097_g1) as housekeeping gene (all from Applied Biosystems, Waltham, MA, USA) according to manufacturer's instructions. Assays were run in MicroAmp Fast 96-well Reaction Plate (0.1 ​ml) (Applied Biosystems, Waltham, MA, USA), using ViiA7 Real-Time PCR System (Applied Biosystems, Waltham, MA, USA). Patient samples were run in single wells and the negative controls in duplicates.

Gene expression is presented as fold change and calculated using the 2^-ΔΔCt formula where the individual values were normalized to the average of the healthy control group.

### 2.5 Confocal microscopy

Explants were snap frozen in optimal cutting temperature (OCT) (Sakura, Tokyo, Japan)maximum 24 ​h after surgery or after incubation for 14 days. They were further sectioned in a cryostat to 12 ​μ m, dried in room temperature for 1 ​h before fixation in acetone and placed in −20 ​°C until use. Mouse sera and avidin was used for blocking before incubation with 5 ​μg/ml biotinylated monoclonal antibody against MMP-13 (Novusbio NBP2–72740B, Centennial, CO, USA) or biotinylated isotype control IgG2a (GeneTex Inc. Irvine, CA, USA) before adding Alexa Fluor 555 conjugate streptavidin (Thermo Fisher Scientific, Waltham, MA, USA). Hoechst 34580 (Life technologies, Carlsbad, CA, USA) was used for nuclei staining. Stained sections were mounted with ProLong Gold antifade reagent (Invitrogen, Waltham, MA, USA) and analyzed using a LSM700 confocal microscope (Zeiss, Stockholm, Sweden).

### 2.6 Nitric oxide

By measuring the stable NO metabolite, nitrite, in the supernatants NO levels were monitored during the two weeks of incubation. Briefly, a microplate adaptation of the Griess assay was used where 50 ​μl of culture supernatants was mixed with 50 ​μl of Griess reagent (1% sulfanilamide in 5% H_3_PO_4_ and 0,1% N-(1-Naphthyl)ethylenediamine dihydrochloride) in a 96 well plate. After 10 ​min incubation at room temperature, the optical density was measured with a CLARIOstar microplate reader (BMG Labtech, Ortenberg, Germany) 540 ​nm. A four parametric logistic standard curve with sodium nitrite in fresh culture medium was used for interpolation to calculate the concentration given in μM NO.

### 2.7 Immunohistochemistry

Frozen explants from day 0, before incubation, were sectioned to 12 ​μm thickness. Human thymus was used as control, sectioned in 12 ​μm thickness and fixed in cold acetone. Thymus tissue samples were obtained from children undergoing corrective cardiac surgery where the thymus is removed to gain access to the heart and all parents gave informed consent. This study was approved by the ethical committee (Dnr 217-12, 2012-04-26). Sections were incubated in PBS, followed by cold 0.3% H_2_O_2_ in PBS before blocking with 2,5% horse serum. Sections were stained with mouse anti-human CD45 at 5 ​μg/ml (clone HI30, Biolegend, San Diego, CA, USA) or IgG1 isotype control (clone MOPC-21, Biolegend, San Diego, CA, USA), followed by ImmPRESS Horse Anti-Mouse IgG Polymer Reagent (ImmPRESS Horse Anti-Mouse IgG PLUS Polymer Kit, MP-7402, Vector laboratories, Burlingame, CA,USA). Staining was developed using ImmPACT AMEC (Vector laboratories, Burlingame, CA, USA), counterstained with Meyer's hematoxylin (VWR, Gothenburg, Sweden) and mounted with a glass coverslip using VectaMount AQ Mounting Medium (Vector laboratories, Burlingame, CA, USA).

### 2.8 Statistics

Statistical differences between day zero samples were calculated using Mann Whitney *U* test. For the kinetic data a mixed model was used with matched values stacked in subcolumns, followed by Tukey's multiple comparisons test. Statistical calculations were performed by GraphPad Prism 8 (GraphPad Software). P ​< ​0.05 was considered statistically significant.

## 3 Results

### 3.1 Spontaneously elevated cytokine levels from RA explants

**Fig. 1:**
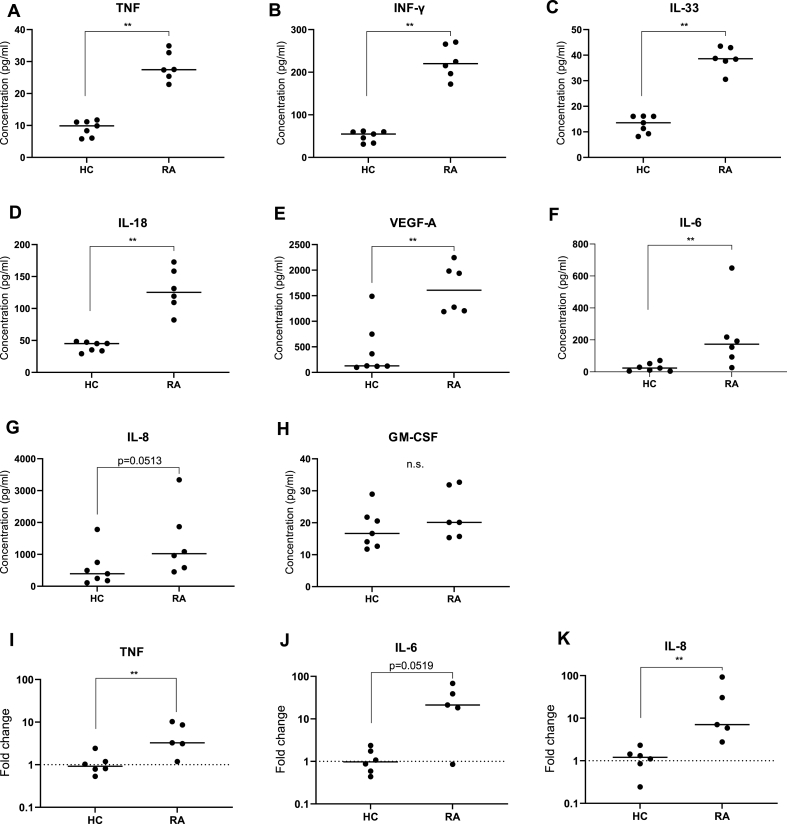
**Cytokine production from unstimulated explants and chondrocytes mRNA expression**. A-H. Cytokine levels in supernatants from HC explants (n ​= ​7) and RA (n ​= ​6) incubated for 16–24 ​h *in vitro* without stimulation measured with LUMINEX. I–K. mRNA expression of TNF, IL-6 and IL-8 from chondrocytes isolated before incubation HC (n ​= ​6) RA (n ​= ​5) measured with qPCR. Rheumatoid arthritis (RA), healthy controls (HC), tumor necrosis factor (TNF), interleukin (IL). Median values from each patient were used and statistical differences were calculated using Mann Whitney. ∗p ​< ​0.05, ∗∗p ​< ​0.01.

To monitor spontaneous release of cytokines from RA and HC explants, the explants were cultured in cultivation medium for 16–24 ​h. The result showed that chondrocytes from both RA and HC explants spontaneously produced several cytokines and that the levels of TNF, INF-γ, IL-33, IL-18, VEGF-A and IL-6 were increased in RA explants compared to HC (Fig. 1A–F). Although the levels of IL-8 did not reach significance (p ​= ​0.05), there was a tendency toward a higher spontaneous production in RA explants with a median value two times higher from RA compared to HC explants (Fig. 1 G). The production of GM-CSF was not statistically different between the groups (Fig. 1 H). To rule out that any leucocytes had infiltrated the cartilage and could contribute to the cytokine production, explants from three RA and three HC were stained for CD45. No positive staining could be seen in either of the explants, compared to thymus tissue that was used as a positive control (Fig S1).

A qPCR could verify that the elevated levels of cytokines were produced by the chondrocytes and not released from the ECM. The mRNA expression of TNF and IL-8 (Fig. 1I, K) was higher in chondrocytes from RA-cartilage compared to HC-cartilage. The same tendency was seen also for the mRNA expression of IL-6 (Fig. 1J).

Taken together, the elevated production of proinflammatory proteins from RA explants compared to HC indicates that RA chondrocytes are primed *in vivo* and posed to produce proinflammatory cytokines. However, it cannot be excluded that some of the proinflammatory cytokines detected could be released from the ECM.

### 3.2 RA explants spontaneously produce high levels of metalloproteases and matrix components

**Fig. 2:**
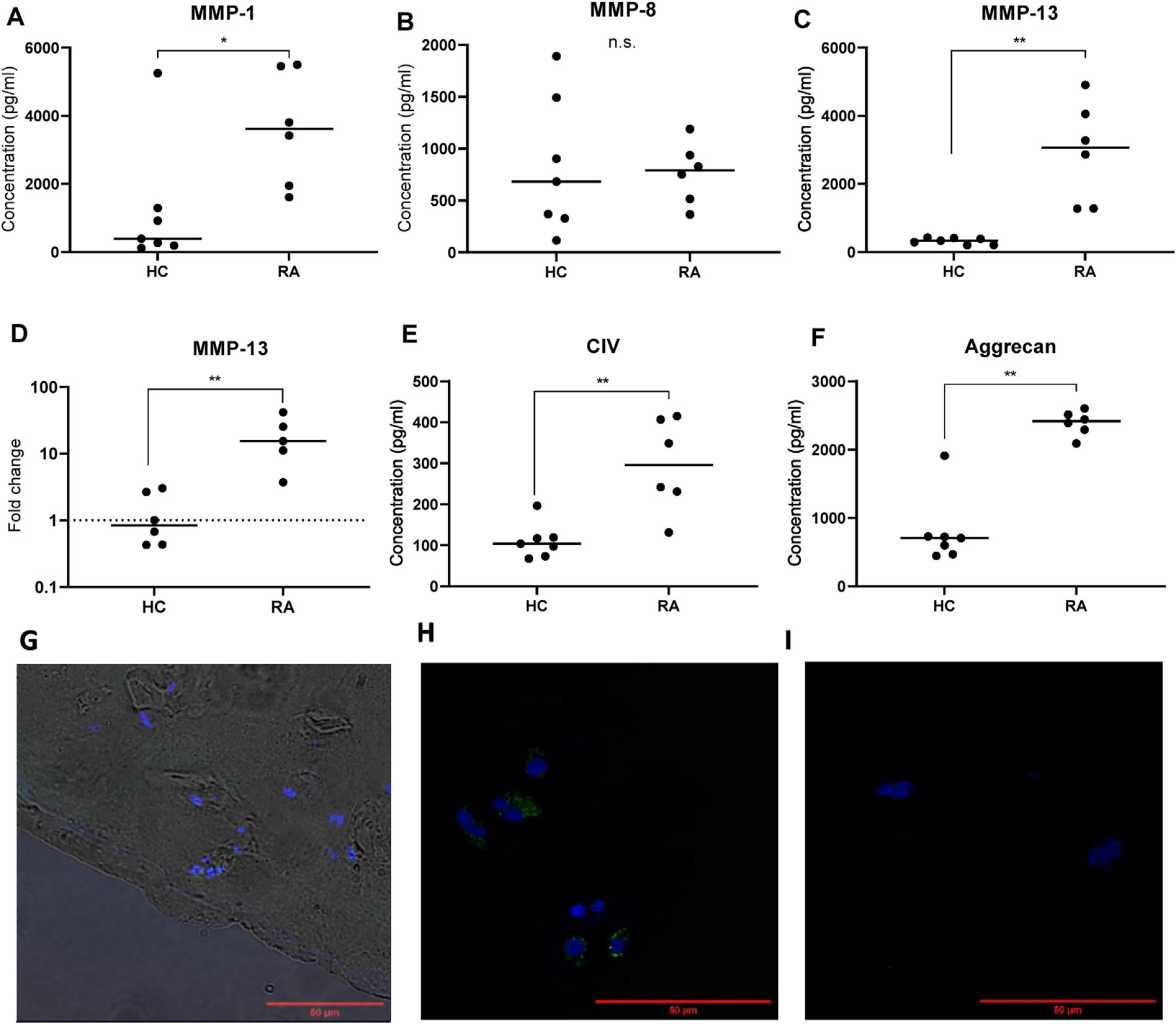
**Metalloproteinase and matrix components produced from unstimulated explants and chondrocytes mRNA expression**. Supernatants from HC (n ​= ​7) and RA (n ​= ​6) explants analyzed for A. MMP-1, B. MMP-8, C. MMP-13 E. collagen IV a1 and F. aggrecan after 16–24 ​h incubation with LUMINEX. D. mRNA expression of MMP-13 from chondrocytes isolated from cartilage before incubation measured with qPCR HC (n ​= ​6) RA (n ​= ​5). Confocal images of cryosections from RA explants stained with biotinylated antibody against MMP-13 or isotype control IgG1 followed by streptavidin conjugate Alexa 55. G. Positive MMP-13 staining in green day 0, before incubation, (40× objective) with a widefield overlay to visualize the MMP-13 located in the chondrons. H. MMP-13 staining day 14 after incubation in medium (60× objective) and I. isotype control IgG1 (60× objective). Scalebar in red indicate 50 ​μm. Rheumatoid arthritis (RA), healthy controls (HC), metalloproteinases (MMP). Median values from each patient were used and statistical differences were calculated using Mann Whitney. ∗p ​< ​0.05, ∗∗p ​< ​0.01.

MMPs have been identified to participate in the pathology of cartilage destruction hence taking part in the proteolytic degradation of ECM [8]. MMP-1 (interstitial collagenase) is mainly involved in the degradation of collagen type I, II, and III and have earlier been described to be constitutively expressed in adult cartilage [11]. MMP-1 was detected in its propeptide form in both RA and HC explants (Fig. 2A), with about 10 times higher levels in supernatants of RA compared to HC explants. There was no difference in the levels MMP-8, detected as both propeptide and mature protein, between RA and HC explants (Fig. 2B) while MMP-13 propeptide showed very similar results to MMP-1 (Fig. 2C). To investigate whether MMPs were stored in matrix and released upon incubation or synthesized and newly released, the MMP-13 mRNA expression from chondrocytes isolated from RA or HC before incubation were analyzed. Further, the mRNA expression of MMP-13 was significantly higher in chondrocytes from RA-cartilage compared with chondrocytes from HC-cartilage (Fig. 2D). This was also confirmed by staining cryosections from RA explants day 0 and 14 and the positive MMP-13 staining was localized within the chondrons of the cartilage (Fig. 2G). These findings show that RA chondrocytes have an elevated and spontaneous production of MMPs.

The levels of ECM components collagen IV a1 and aggrecan were significantly increased from RA explants compared to HC explants after 24 ​h of incubation without stimulation (Fig. 2E–F). Collagen IV a1 is a minor collagen in articular cartilage and only makes up 1% of total collagen in adult articular cartilage. It is mainly located in the pericellular matrix and involved in the attachment and integrity of chondrocytes [12]. Soluble collagen IV a1 has also been shown to promote proliferation of chondrocytes in both healthy and osteoarthritic chondrocytes [13]. The collagen IV a1 levels in the supernatants from RA explants were significantly increased compared to those from HC (Fig. 2E). Aggrecan, the major proteoglycan in articular cartilage, here measured as both intact and cleaved protein, was also significantly increased from RA explants compared to HC explants (Fig. 2F).

### 3.3 Effect of IL-1β stimulation on cytokine production during 14 days

**Fig. 3:**
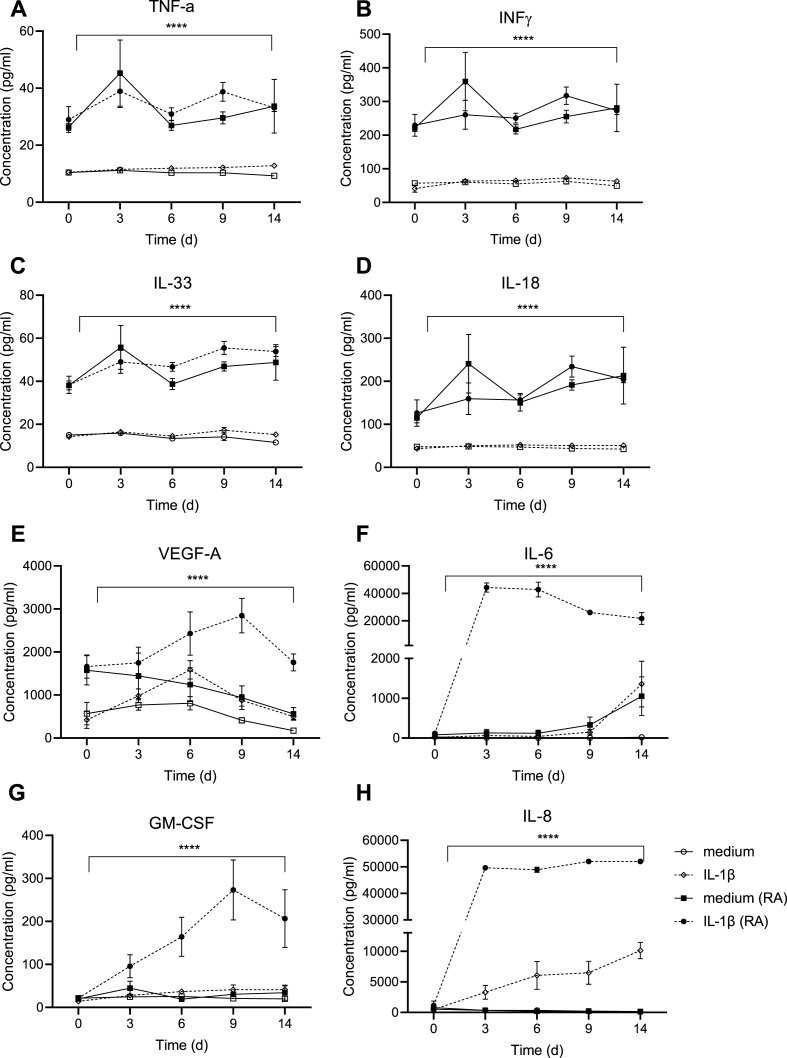
**Cytokines measured after IL-1β stimulation.** Cytokine levels were measured in supernatants from healthy controls (HC) (white, n ​= ​7) and rheumatoid arthritis (RA) (black, n ​= ​6) explants incubated with (broken line) and without interleukin (IL)-1β (unbroken line) harvested at day 0, 3, 6, 9, 14. Mean values ​± ​SEM are shown for each time point and statistical differences were calculated using Mixed effect analysis, comparing the different groups. ∗∗∗∗p ​< ​0.0001.

After assessment of the spontaneous protein levels from RA and HC explants, we evaluated protein expression during 14 days of stimulation with or without IL-1β stimulation. During this time period different kinetic patterns were observed. The cytokines TNF, IFNγ, IL-33 and IL-18 were increased in RA compared to HC explants irrespective of IL-1β stimulation (Fig. 3A–D). VEGF-A showed a different pattern. Stimulation of RA explants with IL-1β significantly increased levels of VEGF-A during the first 9 days where after it declined (Fig. 3E). At the same time, the VEGF-A levels from unstimulated HC and RA explants declined over time. IL-1β stimulation of HC explants did not differ significantly from those that were unstimulated. RA explants responded to IL-1β stimulation with a significant and swift increase in IL-6 compared to unstimulated samples and HC explants. The levels of IL-6 from the IL-1β then remained mostly stable from day 3 until day 14 while the RA unstimulated sample and the HC IL-1β stimulate sample showed an increase at day 14 compared to day 9. (Fig. 3G). GM-CSF levels from RA explants also rose significantly after IL-1β stimulation compared to unstimulated samples and HC explants, although with a delayed kinetics (Fig. 3H). IL-8 levels increased by IL-1β stimulation in both RA and HC explants while both unstimulated samples remained stable during the culture.

### 3.4 Effect of IL-1β stimulation during 14 days on matrix components and metalloproteases

**Fig. 4:**
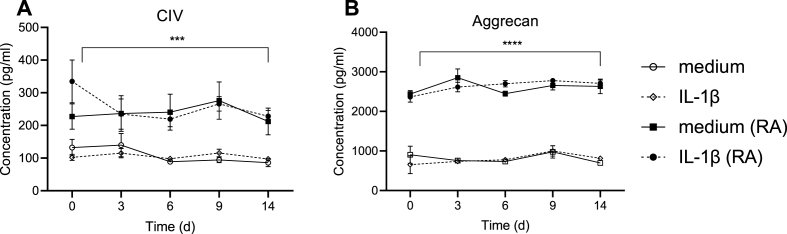
**Extra cellular matrix components measured after IL-1β stimulation.** Levels were measured at day 0, 3, 6, 9, 14 in supernatants harvested from healthy controls (HC) and rheumatoid arthritis (RA) explants incubated with (broken line) and without interleukin (IL)-1β (unbroken line). Duplicate explants were incubated for each stimulation and supernatants were pooled before analyzed, n ​= ​7 (HC, white) n ​= ​6 (RA, black). Mean values ​± ​SEM are shown for each time point and statistical differences were calculated using Mixed effect analysis, comparing the different groups. ∗∗∗∗p ​< ​0.0001.

The release of matrix components collagen IV a1 and aggrecan were significantly and constantly higher from RA compared to HC explants, independently of IL-1β stimulation (Fig. 4A–B).

**Fig. 5:**
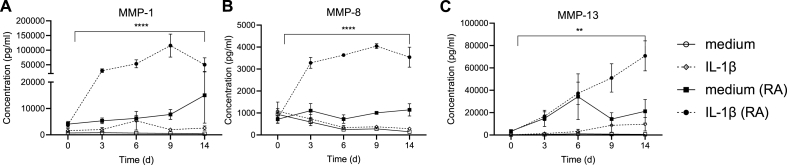
Metalloproteases measured after IL-1β stimulation. *Levels of metalloproteases, (MMPs) were measured at day 0, 3, 6, 9, 14 in supernatants harvested from HC and RA explants incubated with (broken line) and without interleukin (IL)-1β (unbroken line). Duplicate explants were incubated for each stimulation and supernatants were pooled before analyzed, n ​= ​7 (HC, white) n ​= ​6 (RA, black). Rheumatoid arthritis (RA), healthy controls (HC). Mean values* ​± ​*SEM are shown for each time point and statistical differences were calculated using Mixed effect analysis, comparing the different groups. ∗∗∗∗p ​< ​0.0001, ∗∗p ​< ​0.01*.

Metalloproteases showed a different pattern. The release of MMP-1, MMP-8 and MMP-13 were all enhanced by IL-1β stimulation in RA but not in HC explants (Fig. 5). MMP-1 levels in unstimulated RA explants increased over time. HC explants stimulated with IL-1β showed a slight increasing of MMP-1, reaching a peak at day 6 after which it declined (Fig. 5A). MMP-8 showed no differences in HC explants treated with IL-1β compared to medium (Fig. 5B) while MMP-13 could be triggered giving 10 times more MMP-8 compared to unstimulated (Fig. 5C). In summary, our initial observation that RA explants produce higher levels of both ECM components and metalloproteases remained true during two weeks’ observation indicating that the *in vivo* triggered chondrocytes in RA cartilage have a stable phenotype even if removed from the inflammatory milieu. Further, IL-1β stimulation proved to be able to affect metalloproteases levels in RA chondrocytes most effectively but had no effect on the measured ECM components in either RA or HC.

### 3.5 Nitric oxide can be triggered by IL-1β in both healthy and RA chondrocytes

**Fig. 6:**
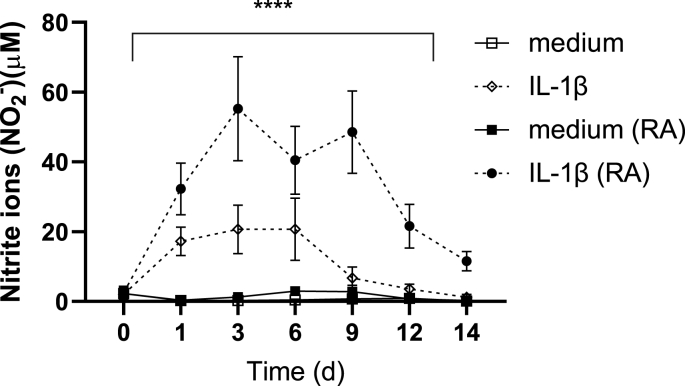
Production of nitric oxide in IL-1β stimulated explants. *RA and HC explants were stimulated with (broken line) and without IL-1β (unbroken line). Duplicate explants were incubated for each stimulation and supernatants were harvested day 0, 1, 3, 6, 9, 12, 14 and pooled.****Nitric oxide****(*NO) concentration was measured indirectly as nitrite (NO_2_^−^) *n ​= ​9 (healthy controls, HC, white) n ​= ​9 (rheumatoid arthritis, RA, black). Mean values* ​± ​*SEM are shown for each time point and statistical differences were calculated using Mixed effect analysis, comparing the different groups. ∗∗∗∗p ​< ​0.0001*.

Chondrocytes have been shown to express inducible nitric oxide synthase (iNOS) through which they can produce NO in response to IL-1β, TNF and bacterial lipopolysaccharides [14]. Elevated levels of NO have been associated with both osteoarthritis and RA. We compared NO levels released from explants from RA and HC that were incubated with and without IL-1β (Fig. 6). All samples showed low, but detectable levels of NO before stimulation at day 0. Further, no significant differences could be seen between RA and HC explants regarding NO levels in unstimulated explants during two weeks’ incubation. However, both RA and HC explants showed an increased production of NO when stimulated with IL-1β with significantly higher levels of NO in RA compared to HC. In summary, explants from both RA and HC produce NO, and the production is increased by IL-1β.

## 4 Discussion

In line with our hypothesis, we show that chondrocytes from RA cartilage explants spontaneously produce higher levels of cytokines and MMPs compared to explants from HC. We also demonstrate that the initial levels of most analytes remain stable over two weeks incubation *ex vivo* indicating that the proinflammatory profile in RA chondrocytes is long lasting. Stimulation with IL-1β increased production of both cytokines and MMPs from RA, but not from HC explants. These findings suggest that chondrocytes contribute to the chronic inflammation in RA joints.

Most studies on cartilage are performed in animal models or in monolayer cultures, most often with chondrocytes from OA patients. Chondrocytes in monolayer cultures quickly dedifferentiate into a more fibroblast like phenotype and the production of cartilage EMC including collagen type II, XI and IX and aggrecan is diminished and collagen type I is increased, which makes a monolayer system unreliable [[15], [16], [17]]. Culture of chondrocytes in explants preserves the 3D structure and diminishes the risk for dedifferentiation, but also put an element of discrepancy in studies using patient material since both the degree of destruction and number of chondrocytes might vary within the same joint [18]. To overcome this hurdle, we have used multiple explants obtained from different areas from the same individual.

In RA, peripheral cartilaginous joints are major targets of the chronic inflammatory process and multiple previous studies have found that proinflammatory cytokines such as IL-1β, TNF and IL-6 are elevated in synovial fluid of RA patients compare to other arthritic lesions such as OA [[19], [20], [21]]. Although the major source of these cytokines has been considered to be synovial fibroblasts as well as infiltrating leucocytes, both ours and others’ results [22] suggest that also the chondrocytes themselves could produce proinflammatory proteins and MMPs. However, many of these studies are performed in monolayer cultures or using animal chondrocytes [22].

As the aim of this study was to determine the proinflammatory activity in chondrocytes from RA patients, we used freshly cultured explants from these patients and compared those to HC. We found an increased production of TNF, INF-γ, IL-33, IL-18, VEGF-A and IL-6 from RA-explants that was sustained up to 14 days. Stimulation with IL-1β led to a substantially increased of VEGF-A, IL-6, GM-CSF and IL-8 over 14 days from RA-explants, but not from the HC. In animal models it has been shown that there is a crosstalk between chondrocytes and fibroblasts that contribute to arthritic joint destruction [23,24]. It is thus very likely that the same situation is in place also in the RA-joints and that the production of proinflammatory cytokines becomes a vicious circle as they enhance each other. In addition to increased inflammation, the chondrocytes also facilitate angiogenesis that supports development of synovitis in the inflamed joint through production of IL-8 [25] and VEGF-A [26]. Both these cytokines are induced by IL-6, one of the main treatment targets in RA [27]. However, the role of IL-6 in cartilage destruction is unclear. In OA patients, IL-6 has been suggested to be associated with cartilage repair [[28], [29], [30]]. Intraarticular injection in arthritis and OA patients with TNF- [31] or IL-1 inhibition [32] have been studied although with limited success, possibly due to toxic effects on the chondrocytes [33].

The proinflammatory cytokines in the joints stimulate the local production of MMPs and aggrecanases, which can degrade all components of the cartilage ECM, for instance during inflammations such as OA and RA [34] Mehana, 2019 #285; [22] The cellular origin and properties of different MMPs vary, and those that mainly have been implicated as produced by chondrocytes are MMP-1, -3 and -13. Except for its ability to degrade the cartilage, MMP-1 has been discussed in relation to bone erosions [[35], [36], [37], [38]] and MMP-3 has been referred to as a biomarker prognosis and treatment responses in RA [39]. MMP-13 is one of the most studied MMPs, and considered as one of the driving factors in OA [40,41], and MMP-13 also resides in the ECM [42]. Indeed, loss of aggrecan, a high molecular weight protein with a proteoglycan core to which long chains of glycosaminoglycan (GAG) are attached, and proteoglycan are considered an early marker of cartilage damage in both RA and OA and associated with MMP production ^34,40.^. Also NO can induce MMP activity [43] in response to cytokines such as TNF and IL-1β, especially the later as it upregulates iNOS in OA chondrocytes [44].

In our study, we could detect protein levels of MMP-1 and -13 from human primary chondrocytes from both HC and RA-patients. Chondrocytes from RA patients responded with a sustained increase of MMP-1 and -13 levels after IL-1β stimulation over time and the synthesis of MMP-13 synthesis from chondrocytes could be confirmed by both confocal microscopy and qPCR. The findings coincided with increased production of both aggrecan and CIV, however whether this is due to novel synthesis or a result of increased protease activity, or a combination of both is not clear. These results suggests that also in human chondrocytes from RA patients there is an ongoing production of cytokines and MMPs that is sustainable over time and that most likely contribute to the local inflammation.

Limitations of this study: The sample size is small, due to the improved treatment of RA during the last decades and reduced need of joint surgery. This has made it difficult to obtain a large number of samples. The degree of destruction and the number of chondrocytes are not evenly distributed in the cartilage. To overcome this hurdle, we have used multiple explants from different areas to ensure as representative material as possible.

The novel treatment strategies for RA that most often includes methotrexate in a combination with a biologic disease modifying anti-rheumatic drug have been very successful, however with increased risks for infections. Significant efforts have also been made to target MMPs in conditions such as OA, cancer and cardiovascular diseases, albeit with limit success [45]. To further develop new and better treatment strategies for patients with RA, it is important to understand the inflammatory crosstalk in the joint, the function and sensibility of the local cells, which not includes not only the synovial tissue but also the chondrocytes.

In conclusion, chondrocytes in RA-patients are activated and produce both cytokines and MMPs spontaneously and during long term stimulation, including both TNF and IL-6 that are main target molecules in RA. Our findings imply that the chondrocytes constitute a local source of proinflammatory molecules and thus could provide an important novel treatment target.

## Contributions

KÖ (Study design; acquisition, Formal analysis and interpretation of data. Drafted the work and substantially revised it, final approval). SH (acquisition, Formal analysis and interpretation of data, revision of the manuscript, final approval). ASH (acquisition, revision of the manuscript, final approval). SA (acquisition, Formal analysis and interpretation of data, revision of the manuscript, final approval)EL (interpretation of data, revision of the manuscript, final approval). CH (acquisition, Formal analysis and interpretation of data, revision of the manuscript, final approval). RH (analysis and interpretation of data. Drafted the work and substantially revised it, final approval). IG (Study design, Formal analysis and interpretation of data. Drafted the work and substantially revised it, final approval).

## Role of the funding source

The project was supported by grants from 10.13039/501100004359Swedish Research Council, 10.13039/501100007857Stiftelsen Konung Gustaf V:s 80-årsfond, 10.13039/501100003849IngaBritt och Arne Lundbergs Forskningsstiftelse, Wilhelm and Martina Lundgrens Vetenskapsfond, Adlerbertska Forskningsstiftelsen, 10.13039/100008448Rune och Ulla Amlövs stiftelse, 10.13039/501100014197Stiftelsen Mary von Sydows donationsfond, 10.13039/100009479Kungl. Vetenskaps-och Vitterhets-Samhället i Göteborg. None of the sponsors were involved in the creation of the study or the manuscript.

## Declaration of competing interest

The authors state no conflict of interest.

## References

[1] Smolen J.S., Aletaha D. (2015). Rheumatoid arthritis therapy reappraisal: strategies, opportunities and challenges. Nat. Rev. Rheumatol..

[2] Goldring M.B., Marcu K.B. (2009). Cartilage homeostasis in health and rheumatic diseases. Arthritis Res. Ther..

[3] Vuolteenaho K., Moilanen T., Knowles R.G., Moilanen E. (2007). The role of nitric oxide in osteoarthritis. Scand. J. Rheumatol..

[4] Lotz M. (2001). Cytokines in cartilage injury and repair. Clin. Orthop. Relat. Res..

[5] Sophia Fox A.J., Bedi A., Rodeo S.A. (2009). The basic science of articular cartilage: structure, composition, and function. Sports Health.

[6] Verzijl N., DeGroot J., Thorpe S.R., Bank R.A., Shaw J.N., Lyons T.J. (2000). Effect of collagen turnover on the accumulation of advanced glycation end products. J. Biol. Chem..

[7] Maroudas A., Bayliss M.T., Uchitel-Kaushansky N., Schneiderman R., Gilav E. (1998). Aggrecan turnover in human articular cartilage: use of aspartic acid racemization as a marker of molecular age. Arch. Biochem. Biophys..

[8] Murphy G., Lee M.H. (2005). What are the roles of metalloproteinases in cartilage and bone damage?. Ann. Rheum. Dis..

[9] Goldring M.B., Otero M., Tsuchimochi K., Ijiri K., Li Y. (2008). Defining the roles of inflammatory and anabolic cytokines in cartilage metabolism. Ann. Rheum. Dis..

[10] Tsuchida A.I., Beekhuizen M., t Hart M.C., Radstake T.R., Dhert W.J., Saris D.B. (2014). Cytokine profiles in the joint depend on pathology, but are different between synovial fluid, cartilage tissue and cultured chondrocytes. Arthritis Res. Ther..

[11] Chubinskaya S., Kuettner K.E., Cole A.A. (1999). Expression of matrix metalloproteinases in normal and damaged articular cartilage from human knee and ankle joints. Lab. Invest..

[12] Eyre D.R., Weis M.A., Wu J.J. (2006). Articular cartilage collagen: an irreplaceable framework?. Eur. Cell. Mater..

[13] Smeriglio P., Dhulipala L., Lai J.H., Goodman S.B., Dragoo J.L., Smith R.L. (2015). Collagen VI enhances cartilage tissue generation by stimulating chondrocyte proliferation. Tissue Eng..

[14] Stadler J., Stefanovic-Racic M., Billiar T.R., Curran R.D., McIntyre L.A., Georgescu H.I. (1991). Articular chondrocytes synthesize nitric oxide in response to cytokines and lipopolysaccharide. J. Immunol..

[15] Malicev E., Kregar-Velikonja N., Barlic A., Alibegović A., Drobnic M. (2009). Comparison of articular and auricular cartilage as a cell source for the autologous chondrocyte implantation. J. Orthop. Res. : official publication of the Orthopaedic Research Society.

[16] Schulze-Tanzil G. (2009). Activation and dedifferentiation of chondrocytes: implications in cartilage injury and repair. Ann. Anat. = Anatomischer Anzeiger.

[17] Duan L., Ma B., Liang Y., Chen J., Zhu W., Li M. (2015). Cytokine networking of chondrocyte dedifferentiation in vitro and its implications for cell-based cartilage therapy. Am. J. Transl. Res..

[18] Fukui N., Miyamoto Y., Nakajima M., Ikeda Y., Hikita A., Furukawa H. (2008). Zonal gene expression of chondrocytes in osteoarthritic cartilage. Arthritis Rheum..

[19] Hampel U., Sesselmann S., Iserovich P., Sel S., Paulsen F., Sack R. (2013). Chemokine and cytokine levels in osteoarthritis and rheumatoid arthritis synovial fluid. J. Immunol. Methods.

[20] Lettesjö H., Nordström E., Ström H., Nilsson B., Glinghammar B., Dahlstedt L. (1998). Synovial fluid cytokines in patients with rheumatoid arthritis or other arthritic lesions. Scand. J. Immunol..

[21] Santos Savio A., Machado Diaz A.C., Chico Capote A., Miranda Navarro J., Rodríguez Alvarez Y., Bringas Pérez R. (2015). Differential expression of pro-inflammatory cytokines IL-15Ralpha, IL-15, IL-6 and TNFalpha in synovial fluid from rheumatoid arthritis patients. BMC Muscoskel. Disord..

[22] Tseng C.C., Chen Y.J., Chang W.A., Tsai W.C., Ou T.T., Wu C.C. (2020). Dual role of chondrocytes in rheumatoid arthritis: the chicken and the egg. Int. J. Mol. Sci..

[23] Steenvoorden M.M., Bank R.A., Ronday H.K., Toes R.E., Huizinga T.W., DeGroot J. (2007). Fibroblast-like synoviocyte-chondrocyte interaction in cartilage degradation. Clin. Exp. Rheumatol..

[24] Korb-Pap A., Stratis A., Muhlenberg K., Niederreiter B., Hayer S., Echtermeyer F. (2012). Early structural changes in cartilage and bone are required for the attachment and invasion of inflamed synovial tissue during destructive inflammatory arthritis. Ann. Rheum. Dis..

[25] Koch A.E., Volin M.V., Woods J.M., Kunkel S.L., Connors M.A., Harlow L.A. (2001). Regulation of angiogenesis by the C-X-C chemokines interleukin-8 and epithelial neutrophil activating peptide 78 in the rheumatoid joint. Arthritis Rheum..

[26] Connolly D.T., Heuvelman D.M., Nelson R., Olander J.V., Eppley B.L., Delfino J.J. (1989). Tumor vascular permeability factor stimulates endothelial cell growth and angiogenesis. J. Clin. Invest..

[27] Srirangan S., Choy E.H. (2010). The role of interleukin 6 in the pathophysiology of rheumatoid arthritis. Ther. Adv. Musculoskelet Dis..

[28] Tsuchida A.I., Beekhuizen M., Rutgers M., van Osch G.J., Bekkers J.E., Bot A.G. (2012). Interleukin-6 is elevated in synovial fluid of patients with focal cartilage defects and stimulates cartilage matrix production in an in vitro regeneration model. Arthritis Res. Ther..

[29] Silacci P., Dayer J.M., Desgeorges A., Peter R., Manueddu C., Guerne P.A. (1998). Interleukin (IL)-6 and its soluble receptor induce TIMP-1 expression in synoviocytes and chondrocytes, and block IL-1-induced collagenolytic activity. J. Biol. Chem..

[30] Yamagata K., Nakayamada S., Zhang T., Zhang X., Tanaka Y. (2020). Soluble IL-6R promotes chondrogenic differentiation of mesenchymal stem cells to enhance the repair of articular cartilage defects using a rat model for rheumatoid arthritis. Clin. Exp. Rheumatol..

[31] Bello S., Bonali C., Serafino L., Rotondo C., Terlizzi N., Lapadula G. (2013). Intra-articular therapy with tumor necrosis factor-α antagonists: an update. Reumatismo.

[32] Chevalier X., Goupille P., Beaulieu A.D., Burch F.X., Bensen W.G., Conrozier T. (2009). Intraarticular injection of anakinra in osteoarthritis of the knee: a multicenter, randomized, double-blind, placebo-controlled study. Arthritis Rheum..

[33] Guzelant A.Y., Isyar M., Yilmaz I., Sirin D.Y., Cakmak S., Mahirogullari M. (2017). Are chondrocytes damaged when rheumatologic inflammation is suppressed?. Drug Chem. Toxicol..

[34] Otero M., Goldring M.B. (2007). Cells of the synovium in rheumatoid arthritis. Chondrocytes. Arthritis Res. Ther..

[35] Cunnane G., Fitzgerald O., Beeton C., Cawston T.E., Bresnihan B. (2001). Early joint erosions and serum levels of matrix metalloproteinase 1, matrix metalloproteinase 3, and tissue inhibitor of metalloproteinases 1 in rheumatoid arthritis. Arthritis Rheum..

[36] Cunnane G., FitzGerald O., Hummel K.M., Youssef P.P., Gay R.E., Gay S. (2001). Synovial tissue protease gene expression and joint erosions in early rheumatoid arthritis. Arthritis Rheum..

[37] Murphy E., Roux-Lombard P., Rooney T., Fitzgerald O., Dayer J.M., Bresnihan B. (2009). Serum levels of tissue inhibitor of metalloproteinase-1 and periarticular bone loss in early rheumatoid arthritis. Clin. Rheumatol..

[38] Ita M.E., Ghimire P., Welch R.L., Troche H.R., Winkelstein B.A. (2020). Intra-articular collagenase in the spinal facet joint induces pain, DRG neuron dysregulation and increased MMP-1 absent evidence of joint destruction. Sci. Rep..

[39] Lerner A., Neidhofer S., Reuter S., Matthias T. (2018). MMP3 is a reliable marker for disease activity, radiological monitoring, disease outcome predictability, and therapeutic response in rheumatoid arthritis. Best Pract. Res. Clin. Rheumatol..

[40] Rose B.J., Kooyman D.L. (2016). A tale of two joints: the role of matrix metalloproteases in cartilage biology. Dis. Markers.

[41] Li H., Wang D., Yuan Y., Min J. (2017). New insights on the MMP-13 regulatory network in the pathogenesis of early osteoarthritis. Arthritis Res. Ther..

[42] Mehana E.E., Khafaga A.F., El-Blehi S.S. (2019). The role of matrix metalloproteinases in osteoarthritis pathogenesis: an updated review. Life Sci..

[43] Murrell G.A.C., Jang D., Williams R.J. (1995). Nitric axide activates metalloprotease enzymes in articular cartilage. Biochem. Biophys. Res. Commun..

[44] Vuolteenaho K., Moilanen T., Al-Saffar N., Knowles R., Moilanen E. (2001). Regulation of the nitric oxide production resulting from the glucocorticoid-insensitive expression of iNOS in human osteoarthritic cartilage. Osteoarthritis Cartilage.

[45] Malemud C.J. (2019). Inhibition of MMPs and ADAM/ADAMTS. Biochem. Pharmacol..

