# Supplementary material for: Rheumatoid arthritis chondrocytes produce increased levels of pro-inflammatory proteins
Source: Osteoarthr Cartil Open. 2022 Jan 21;4(1):100235. doi: 10.1016/j.ocarto.2022.100235 (PMC9718183; doi:10.1016/j.ocarto.2022.100235)
Supplement: Multimedia component 1 [file mmc1.docx]

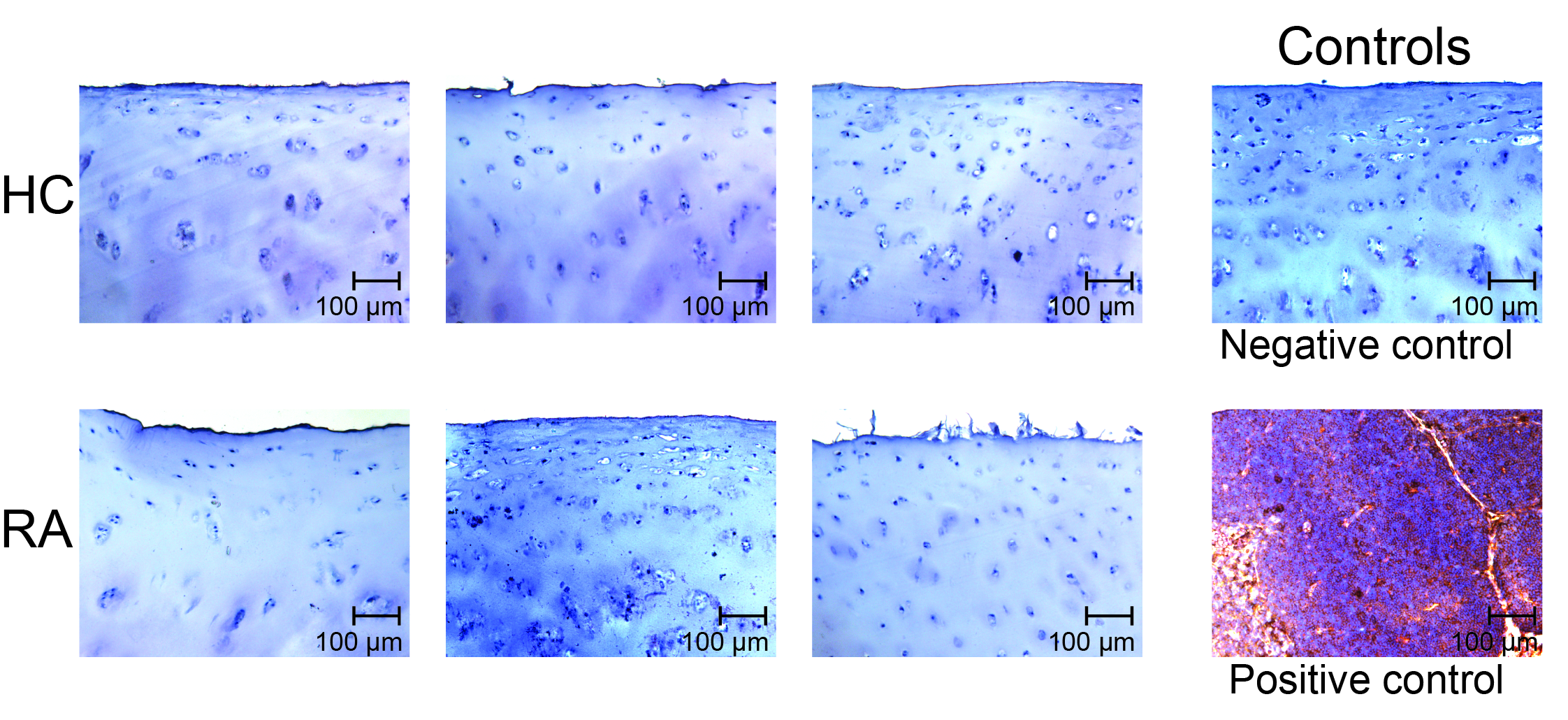
Sup. Fig. 1

***Supplementary figure 1. CD45 staining.*** *Immunohistochemistry showing CD45 staining on cryosections from HC and RA explants (d0, n=3). An isotype IgG control antibody was used as a negative control (top right image) while human thymus tissue was used as a positive control (lower right image). Rheumatoid arthritis (RA), healthy controls (HC).*
